# De novo *PHIP*-predicted deleterious variants are associated with developmental delay, intellectual disability, obesity, and dysmorphic features

**DOI:** 10.1101/mcs.a001172

**Published:** 2016-11

**Authors:** Emily Webster, Megan T. Cho, Nora Alexander, Sonal Desai, Sakkubai Naidu, Mir Reza Bekheirnia, Andrea Lewis, Kyle Retterer, Jane Juusola, Wendy K. Chung

**Affiliations:** 1 Department of Pediatrics, Columbia University Medical Center, New York, New York 10032, USA;; 2 GeneDx, Gaithersburg, Maryland 20877, USA;; 3 Kennedy Krieger Institute, Baltimore, Maryland 21205, USA;; 4 Department of Molecular and Human Genetics, Baylor College of Medicine, Houston, Texas 77030, USA;; 5 Department of Medicine, Columbia University Medical Center, New York, New York 10032, USA

**Keywords:** abdominal obesity, central hypotonia, intellectual disability, mild, mild global developmental delay

## Abstract

Using whole-exome sequencing, we have identified novel de novo heterozygous pleckstrin homology domain-interacting protein (*PHIP*) variants that are predicted to be deleterious, including a frameshift deletion, in two unrelated patients with common clinical features of developmental delay, intellectual disability, anxiety, hypotonia, poor balance, obesity, and dysmorphic features. A nonsense mutation in *PHIP* has previously been associated with similar clinical features. Patients with microdeletions of 6q14.1, including *PHIP*, have a similar phenotype of developmental delay, intellectual disability, hypotonia, and obesity, suggesting that the phenotype of our patients is a result of loss-of-function mutations. *PHIP* produces multiple protein products, such as PHIP1 (also known as DCAF14), PHIP, and NDRP. PHIP1 is one of the multiple substrate receptors of the proteolytic CUL4-DDB1 ubiquitin ligase complex. CUL4B deficiency has been associated with intellectual disability, central obesity, muscle wasting, and dysmorphic features. The overlapping phenotype associated with CUL4B deficiency suggests that *PHIP* mutations cause disease through disruption of the ubiquitin ligase pathway.

## INTRODUCTION

Pleckstrin homology domain-interacting protein (*PHIP*; ENSG00000146247), located on Chromosome 6q14.1, was identified as a candidate gene for severe intellectual disability in one child in a study of 100 children with intelligence quotients (IQs) below 50 and their unaffected parents (de Ligt et al. 2012). *PHIP* produces at least three proteins (PHIP1, PHIP, and NDRP) through alternative splicing. PHIP1 (also known as DCAF14) acts as a substrate receptor in a ubiquitin ligase pathway and mediates substrate-specific proteolysis (Lee and Zhou 2007). Mutations affecting ubiquitin ligase pathways have been associated with a number of diseases, including neurological disorders, autoimmune disorders, and cancers (Ardley and Robinson 2004; Matentzoglu and Scheffner 2008; Lohr et al. 2010; Lipkowitz and Weissman 2011). Using whole-exome sequencing (WES), we identified two novel de novo heterozygous predicted deleterious *PHIP* variants in two unrelated patients with a common phenotype of developmental delay, intellectual disability, anxiety, hypotonia, poor balance, obesity, and dysmorphic features.

## RESULTS

### Genomic Analysis

A total of 2522 patients referred to a single clinical genetic diagnostic lab (GeneDx) with developmental delay or intellectual disability were analyzed by WES. Two patients were identified with novel de novo variants in *PHIP*. WES of the two patients identified a de novo variant in the *PHIP* gene produced an average of 9.3 GB of sequence per sample. The mean coverage of captured regions was ∼120× per sample, with ∼98% covered with at least 10× coverage, an average of 92% of base call quality of Q30 or greater and an overall average mean quality score of Q36 (Supplemental Table S1).

The variants found in *PHIP* include a frameshift deletion, NM_017934.5:p.L260Wfs*48, and a missense variant, NM_017934.5:p.F17S, located in an α-helix (Table 1). Neither of the variants has been observed in ExAC (Exome Aggregation Consortium; http://exac.broadinstitute.org, accessed in September 2015). Predictions of pathogenicity for p.F17S were variable, but the variant was predicted to be pathogenic by SIFT (Sorting Intolerant from Tolerant; Kumar et al. 2009), PROVEAN (Protein Variation Effect Analyzer; Choi and Chan 2015), MutationTaster (Schwarz et al. 2014), and CADD (Combined Annotation-Dependent Depletion; Kircher et al. 2014). Cross-species comparison suggests that the phenylalanine at position 17 is highly conserved. *PHIP* has a high haploinsufficiency score [*p*(HI) = 0.95] (Huang et al. 2010), as well as intolerance to both loss-of-function variants [(*p*(LI) = 1.00] and missense variants (*z*-score = 5.20) (Exome Aggregation Consortium et al. 2015).

**Table 1.** Variant table

Gene	Chromosome	NCBI reference sequence	HGVS DNA reference	HGVS protein reference	Variant type	Predicted effect	dbSNP/dbVar ID	Genotype
*PHIP*	6q14.1	NM_017934.5	c.50T>C	p.F17S	Missense	Loss of function	None	Heterozygous
*PHIP*	6q14.1	NM_017934.5	c.779delT	p.L260Wfs*48	Frameshift deletion	Loss of function	None	Heterozygous

NCBI, National Center for Biotechnology Information; HGVS, Human Genome Variation Society; dbSNP, Database for Short Genetic Variations; dbVar, Database of Genomic Structural Variation.

### Clinical Presentation

**Figure 1.**
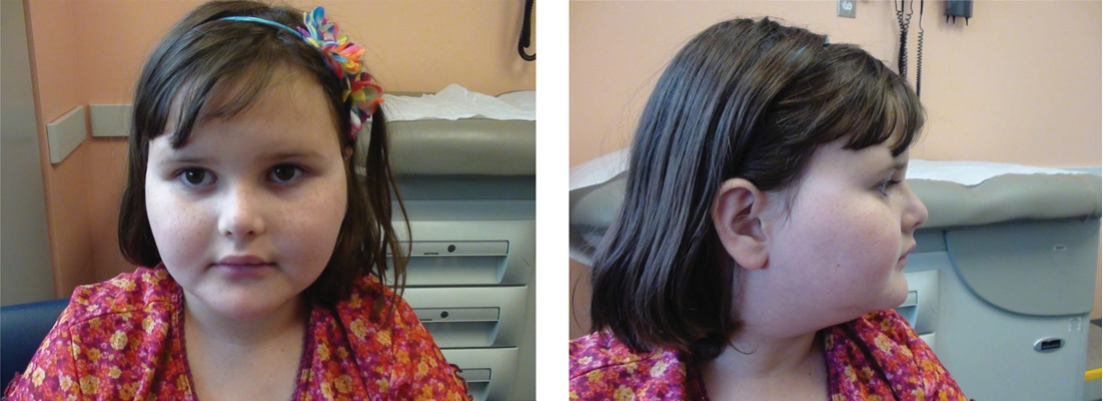
Facial dysmorphisms of Patient 1 showing a small, upturned nose and deep-set eyes.

Both patients with predicted deleterious de novo *PHIP* variants are females, ages 14 (Patient 1) and 8 (Patient 2), and have common features of global developmental delay, intellectual disability, anxiety, hypotonia, poor balance, obesity, and dysmorphic facial features (Table 2; Fig. 1). Both patients had normal weight at birth, but are now at or above the 97th percentile for weight and body mass index (BMI). Brain malformations are not associated with either patient.

**Table 2.** Clinical features of individuals with predicted deleterious variants in *PHIP*

Patient no.	1	2	de Ligt et al. (Trio 5)
Current age (yr)	14	8	UNK
Gender	Female	Female	Female
Variant	c.50T>C:p.F17S	c.779delT:p.L260Wfs*48	c.3447T>G:p.Y1149*
Prenatal issues	N	N	Meconium stained amniotic fluid
Neonatal issues	N	N	Feeding problems
Congenital anomalies	Hip dysplasia	Laryngeal cleft	N
Dysmorphic features	Fleshy ears, full chin, micrognathia	Fleshy earlobes, small nose, deep-set eyes, up-turned upper lip, short and smooth philtrum, round face	Straight eyebrows, blepharophimosis, mild ptosis, long philtrum, full lips, tapered fingers, clinodactyly of the fifth finger, long toes
Birth weight	WT = 3.40 kg (64%)	WT = 3.35 kg (60%)	
Current BMI, HT, WT, OFC	At 12 yr 10 mo: BMI = 29.9 kg/m^2^ (z-score = 2.06) WT = 81.5 kg (99%) HT = 165 cm (89%) OFC = 57 cm (>97%)	At 8 yr 2 mo: BMI = 23.6 kg/m^2^ (z-score = 2.08) WT = 39.8 kg (97%) HT = 129.9 cm (59%) OFC = 49.1 cm (4%)	At UNK age: BMI = 25.4 kg/m^2^ WT = 56.8 kg (>+2 SD) HT = 149.5 cm (+0.5 SD) OFC = 57.6 cm (>+2.5 SD)
Obesity	Y	Y	Y
Insulin resistance	Y	N	
Developmental delay	Y	Y	Y
Age at sitting	9 mo	6 mo	12 mo
Age at walking	18 mo	14 mo	24 mo
Age at talking	18 mo	First words at 16 mo, words together by 3 yr, sentences at 4 yr, could not be understood by most people until age 5	First word at 5 yr
Intellectual disabilities	Y	Y	Y
Full-scale IQ	60	UNK	<50
Current speech abilities	Speaks in full sentences	Nasal speech	
ADD	Y	N	
Anxiety	Y	Y	
Brain MRI/CT results	Unremarkable	Unremarkable	
Behavioral issues	N	Y, aggressive toward siblings	
Regression	N	N	
Hypotonia	Y	Y	
Balance/coordination	Poor	Poor	
Orthopedic issues	N	N	
Gastrointestinal	N	Diarrhea, constipation	
Ophthalmologic issues	N	Strabismus	Strabismus
Self-care abilities (e.g., dressing, feeding)	Began dressing herself at 9 yr, has trouble with buttons/shoes	Can dress, feed, and help herself but hates utensils, prefers her hands, is sloppy with brushing her hair	
Other significant medical problems	Polycystic ovarian disease, menstrual irregularities	Headaches, sleep problems	
Additional genetic variants	5q23.2 duplication, inherited		
Other notes:		Translucent skin on chest/abdomen	

Y, yes; N, no; UNK, unknown; mo, months; yr, years; BMI, body mass index; HT, height; WT, weight; OFC, occipital frontal circumference; %, percentile; ADD, attention-deficit disorder; SD, standard deviations.

Patient 1 has an IQ of 60, which dropped from a previous IQ of 83. In addition to obesity, Patient 1 has insulin resistance and polycystic ovarian syndrome diagnosed by an endocrinologist and treated with metformin. She has dysmorphic features including fleshy ears, full chin, and micrognathia. Before WES, a chromosome microarray was performed that revealed a 95-kb paternally inherited 5q23.2 duplication, including *CEP120M* and *CSNK1G3* genes.

Patient 2 has behavioral issues and is aggressive to her siblings. She also has a number of dysmorphic facial features, including fleshy earlobes, small nose, deep-set eyes, up-turned upper lip, short philtrum, and round face, and had a laryngeal cleft at birth. A chromosome microarray was normal.

## DISCUSSION

Using clinical WES, we have identified two unrelated patients with novel de novo heterozygous predicted deleterious variants in the *PHIP* gene with a common clinical phenotype of developmental delay, intellectual disability, anxiety, hypotonia, poor balance, obesity, and dysmorphic facial features.

The phenotype of our two patients is similar to a previously reported patient with a de novo heterozygous nonsense mutation in *PHIP*, p.Y1149* (c.3447T>G), who has developmental delay, severe intellectual disability (IQ < 50), obesity (weight > +2 SD), and facial dysmorphisms (de Ligt et al. 2012).

Nine patients with microdeletions spanning the 6q14.1 region including the *PHIP* gene have been reported with a similar phenotype including developmental delay, intellectual disability, hypotonia, and obesity (Becker et al. 2012; Wentzel et al. 2010). However, patients with microdeletions encompassing the *PHIP* gene have a number of additional clinical findings that do not overlap with the phenotype of our patients, probably because of the deletion of adjacent genes in 6q14. We suggest that the microdeletion data, our frameshift mutation, and the previously reported patient with a nonsense mutation support an autosomal dominant loss-of-function mechanism for disease caused by mutations in *PHIP*. *PHIP* is predicted to be sensitive to haploinsufficiency (Huang et al. 2010).

**Figure 2.**
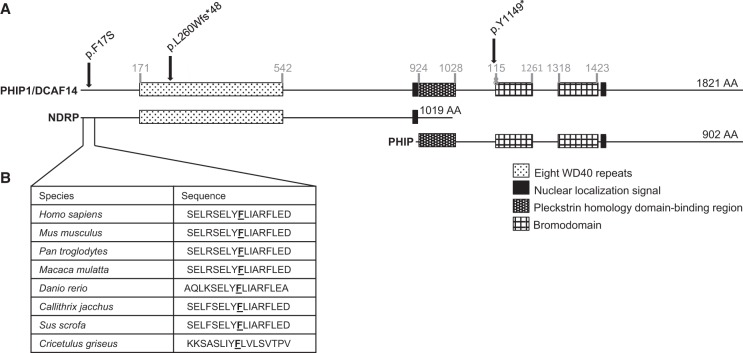
(*A*) Schematic representation of *PHIP* protein products and predicted deleterious variants. *PHIP* produces at least three proteins through alternative splicing: PHIP1 (also known as DCAF14), PHIP, and NDRP. (*B*) *PHIP* sequence conservation across species. Underlined letter in sequences aligns with the p.F17S variant.

*PHIP* produces at least three proteins through alternative splicing: PHIP1 (also known as DCAF14), PHIP, and NDRP (Fig. 2; Farhang-Fallah et al. 2000; Kato et al. 2000; Podcheko et al. 2007). Domains of these proteins include a β-propeller-forming WD40 repeat domain, nuclear localization signals, a pleckstrin homology domain-binding region, and bromodomains (Farhang-Fallah et al. 2000; Podcheko et al. 2007). Notably, PHIP1 is the only protein product disrupted by all three variants. The two predicted deleterious variants we report are in the PHIP1 and NDRP protein-coding region, and the mutation described by de Ligt et al. (2012) falls in the PHIP1 and PHIP protein-coding region. It is possible that the phenotype observed in our patients is due to disruption of PHIP1 alone.

PHIP1, also known as DCAF14 (DDB1- and CUL4-associated factor), is a member of the DCAF protein family (Jin et al. 2006; Lee and Zhou 2007). DCAFs act as substrate receptors for ubiquitin E3 ligases using a CUL4-DDB1 complex (Higa et al. 2006; Higa and Zhang 2007). The CUL4-DBB1 ubiquitin ligase binds to various substrate receptors to target specific proteins for proteolysis and is involved in a variety of regulatory pathways, including gene transcription, cell cycle, cell death, and embryonic development (Higa and Zhang 2007; Lee and Zhou 2007). The substrate(s) and protein targets of the CUL4-DDB1-PHIP1 complex are not yet known.

**Figure 3.**
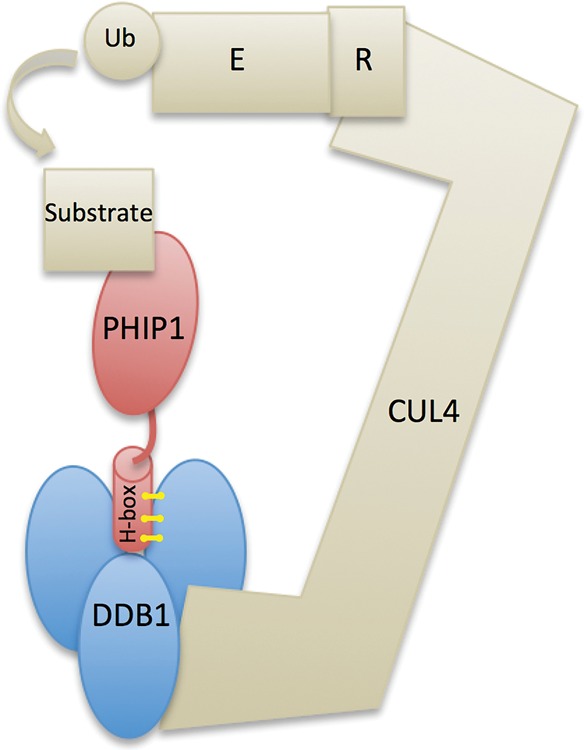
Depiction of the CUL4-DDB1 ubiquitin ligase complex and the role of the predicted PHIP1 H-box. Yellow lines indicate hydrophobic interactions between the PHIP1 H-box and DDB1. Ub, ubiquitin; E, ubiquitin-conjugating enzyme; R, RING finger protein. (Adapted from Lee and Zhou 2007.)

The role of PHIP1 as a substrate receptor for the CUL4-DDB1 complex may explain how the p.F17S variant causes protein dysfunction. At least seven DCAFs have α-helices close to the amino terminus that are important for DCAF-DDB1 binding (Li et al. 2010). This binding motif, termed H-box, is thought to facilitate DCAF-DDB1 interaction in part through hydrophobic interactions (Li et al. 2010). The amino-terminus α-helix of PHIP1, in which the missense variant is located, may function as an H-box (Fig. 3). The substitution of a hydrophobic amino acid, phenylalanine, for a hydrophilic amino acid, serine, could disrupt PHIP1-DDB1 binding.

CUL4, a member of the cullin-RING ubiquitin ligase family, is expressed in mammals as two paralogs, CUL4A and CUL4B (Zhao and Sun 2012). *CUL4B* is encoded on the X Chromosome, and deficiency in males is associated with intellectual disability, seizures, aggressive outbursts, central obesity, muscle wasting, short stature, macrocephaly, and dysmorphic features including brachydactyly, macroglossia, pes cavus, and prominent upper lip (Tarpey et al. 2007; Zhao and Sun 2012). The phenotype of patients with *CUL4B* mutations overlaps with the phenotype of our patients. Because CUL4B and PHIP1 function in the same ubiquitin ligase pathway, the similarities support our suggestion that *PHIP* variants are causative of the phenotype in our patients.

PHIP1 is ubiquitously expressed in mice, with higher levels of expression in brain, pancreatic islet, and skeletal muscle cells (Podcheko et al. 2007). The expression pattern is consistent with the neurologic and metabolic phenotypes in our patients. The obesity in these patients could be the result of disruption of PHIP1 effects on brain-mediated food intake and energetics. The insulin resistance observed could be secondary to obesity or mediated centrally. Through its pleckstrin homology domain-binding region, PHIP interacts with insulin receptor substrate 1 (IRS1) to propagate insulin signaling, leading to insulin-dependent mitogenesis and GLUT4 translocation to the plasma membrane because of cytoskeletal reorganization (Farhang-Fallah et al. 2002). The expression pattern of *Phip1* in mice, the similar phenotype of our two patients, the previously reported patient with a *PHIP* mutation, and the overlapping phenotype of individuals with 6q14.1 microdeletions and *CUL4B* mutations collectively suggest that the mutation in *PHIP* is responsible for the phenotype observed in our patients and that the phenotype is probably caused by disruption of the ubiquitin ligase pathway due to loss of function of PHIP1.

## METHODS

### Whole-Exome Sequencing

Genomic DNA was extracted from whole blood of affected children and their parents. Exome sequencing was performed on exon targets captured using the Agilent SureSelect Human All Exon V4 (50 Mb) kit (Agilent Technologies). The sequencing methodology and variant interpretation protocol has been described previously (Tanaka et al. 2015). Variants were confirmed by Sanger sequencing. The general assertion criteria for variant classification are publicly available on the GeneDx ClinVar submission page (http://www.ncbi.nlm.nih.gov/clinvar/submitters/26957/).

## ADDITIONAL INFORMATION

### Data Deposition and Access

The *PHIP* variants found in this study have been deposited in ClinVar (http://www.ncbi.nlm.nih.gov/clinvar/) under accession numbers SCV000282077 and SCV000282078. Raw WES data could not be deposited because of a lack of patient permission.

### Ethics Statement

This study was approved by the Institutional Review Board of Columbia University. Consent to be part of this study was obtained from both families (verbal consent from the family of Patient 1 and written consent from the family of Patient 2).

### Acknowledgments

We gratefully acknowledge the contributions of the patients and their families.

### Author Contributions

E.W., M.T.C., N.A., and J.J. analyzed the data as well as drafted and critically reviewed the manuscript. S.D., S.N., M.R.B., and A.L. provided the clinical data and critically reviewed the manuscript. K.R. generated and analyzed the data and critically reviewed the manuscript. W.K.C. conceived of the study, analyzed the data, and drafted and critically reviewed the manuscript.

### Funding

This work was supported in part by a grant from the Simons Foundation to W.K.C. and a National Institute of Diabetes and Digestive and Kidney Diseases (NIDDK) T35 training grant (T35DK093430) to E.W.

### Competing Interest Statement

M.T.C., K.R., and J.J. are employees of GeneDx. W.K.C. is a former employee of GeneDx.

## Supplementary Material

Supplemental Material
