# Supplementary material for: De novo PHIP-predicted deleterious variants are associated with developmental delay, intellectual disability, obesity, and dysmorphic features
Source: Cold Spring Harb Mol Case Stud. 2016 Nov;2(6):a001172. doi: 10.1101/mcs.a001172 (PMC5111011; doi:10.1101/mcs.a001172)
Supplement: Supplemental Material [file supp_2_6_a001172__index.html]

De novo PHIP-predicted deleterious variants are associated with developmental delay, intellectual disability, obesity, and dysmorphic features — Supplemental Material 

# De novo *PHIP*-predicted deleterious variants are associated with developmental delay, intellectual disability, obesity, and dysmorphic features

## Supplemental Material

**Files in this Data Supplement:**

- Supp Table 1.docx
