# Supplementary material for: De novo PHIP-predicted deleterious variants are associated with developmental delay, intellectual disability, obesity, and dysmorphic features
Source: Cold Spring Harb Mol Case Stud. 2016 Nov;2(6):a001172. doi: 10.1101/mcs.a001172 (PMC5111011; doi:10.1101/mcs.a001172)
Supplement: Supplemental Material [file supp_mcs.a001172_SuppTable1.docx]

| Patient | 10x Cov | Mean Cov | Yield (Gb) | Q30 | MeanQ | Filtered Vars | *PHIP* Mean CDS Cov | Var Total Fam Cov | Mean Per-Sample Var Cov |
| --- | --- | --- | --- | --- | --- | --- | --- | --- | --- |
| 1 | 98.82% | 144 | 11.4 | 92 | 36 | 5175 | 204 | 128 | 43 |
| 2 | 98.08% | 98 | 7.1 | 91 | 36 | 4624 | 111 | 299 | 100 |
| MEAN | 98.45% | 121 | 9.3 | 92 | 36 | 4900 | 158 | 214 | 71 |

Supplemental Table 1. Sequencing quality metrics

cov., coverage; Gb, Gigabytes; CDS, coding sequence; var., variance; fam, family.
